# Advanced pancreatic cancer: a meta-analysis of clinical trials over thirty years

**DOI:** 10.18632/oncotarget.25036

**Published:** 2018-04-10

**Authors:** Bradley R. Hall, Andrew Cannon, Pranita Atri, Christopher S. Wichman, Lynette M. Smith, Apar K. Ganti, Chandrakanth Are, Aaron R. Sasson, Sushil Kumar, Surinder K. Batra

**Affiliations:** ^1^ Department of Surgery, Division of Surgical Oncology, University of Nebraska Medical Center, Omaha, NE, USA; ^2^ Department of Biochemistry and Molecular Biology, University of Nebraska Medical Center, Omaha, NE, USA; ^3^ Department of Biostatistics, College of Public Health, University of Nebraska Medical Center, Omaha, NE, USA; ^4^ Department of Internal Medicine, Division of Hematology and Oncology, University of Nebraska Medical Center, Omaha, NE, USA; ^5^ Department of Internal Medicine, Division of Hematology and Oncology, VA Nebraska-Western Iowa Health Care System, Omaha, NE, USA; ^6^ Department of Surgery, Division of Surgical Oncology, Stony Brook School of Medicine, Stony Brooke, NY, USA; ^7^ Fred and Pamela Buffett Cancer Center, University of Nebraska Medical Center, Omaha, NE, USA

**Keywords:** pancreatic ductal adenocarcinoma, meta-analysis, metastasis, chemotherapy, survival

## Abstract

**Background:**

In contrast to other cancers, survival rates for pancreatic ductal adenocarcinoma (PDAC) patients have improved but minimally over the past thirty years. The aim of this study was to perform a meta-analysis of clinical trials published since 1986 to determine trends in median overall survival in primarily metastatic PDAC.

**Materials and methods:**

All Phase 2–4 clinical trials published during or after 1986 investigating first-line systemic chemotherapy in metastatic PDAC were included in the meta-analysis. Publications obtained through PubMed and www.ClinicalTrials.gov were cross-referenced to identify additional trials. Trials enrolling fewer than 50% of study participants with metastatic disease were excluded.

**Results:**

Of 19,488 patients enrolled in 151 clinical trials, 84% had metastatic disease and 16% had locally advanced pancreatic cancer. In clinical trials published from 1986 to 2016, the weighted median overall survival (wMOS) increased by 3.0 months. The median wMOS was higher in combination therapy (7.31 months, IQR 5.4 to 8.5) compared to non-gemcitabine, single-agent therapy (4.76 months, IQR 3.5 to 6.0), gemcitabine monotherapy (6.48 months, IQR 5.9 to 7.2), and gemcitabine plus single-agent therapy (7.09 months, IQR 6.3 to 8.2). Of all regimens used in more than one study arm, FOLFIRINOX had the highest wMOS (10.9 months).

**Conclusions:**

Regardless of treatment regimen, survival rates in PDAC have minimally improved over time. Of drugs used in two or more study arms, only FOLFIRINOX has a wMOS greater than ten months. Emphasis should, therefore, be placed on identification of novel targets that promote early diagnosis and intervention.

**Funding:**

The authors on this manuscript are in parts, supported by grants from the National Institutes of Health (EDRN U01 CA200466, SPORE P50 CA127297, R01 CA183459, R21 AA026428 and R01 CA 195586).

## INTRODUCTION

In 1997, Burris and colleagues demonstrated that gemcitabine improved symptoms in patients with metastatic pancreatic adenocarcinoma (mPDAC); more important, it also significantly prolonged survival [1]. Since then, however, survival rates have remained stagnant, and no treatment regimen has been shown to extend median overall survival (MOS) beyond twelve months for patients with mPDAC. PDAC is currently the 12th-most common cancer, but is the third-most common cause of cancer-related mortality in the United States [2, 3]. It is estimated that between the years 2010–2030, incidence of and mortality associated with pancreatic cancer will increase by 105% and 71%, respectively [4]. With 53,670 new cases and 43,090 deaths estimated to occur in 2017, pancreatic cancer will likely become the second-most common cause of cancer-related mortality shortly after 2020 [2, 4].

There have been few advances in the treatment of PDAC, and early stage disease remains undetectable in asymptomatic patients. While research in other cancers has led to the development of effective screening modalities, such as mammography (breast cancer) and colonoscopy (colon cancer), no equivalent screening modalities are yet available for PDAC. Thus, clinicians frequently manage patients with advanced disease who are not candidates for curative approaches.

The persistently low survival rate seen in PDAC is unique among cancers. The 5-year overall survival (5Y-OS) rate has improved for nearly all other cancers; for example, patients with melanoma, breast, and prostate cancer now have 5Y-OS rates greater than 90% [2]. These advancements were in part catalyzed by the work of Mary Lasker and the signing of the National Cancer Act of 1971 (generally viewed as the beginning of the “War on Cancer”) [5]. If treatments do not improve or screening modalities are not developed, mortality of PDAC will increase parallel to anticipated increased incidence.

We performed a meta-analysis with two aims; first, to quantify the change in MOS as reported in clinical trials in mPDAC since 1986; and second, to evaluate the efficacy of treatment regimens in these clinical trials.

## RESULTS

### Overview of clinical trials

**Figure 1:**
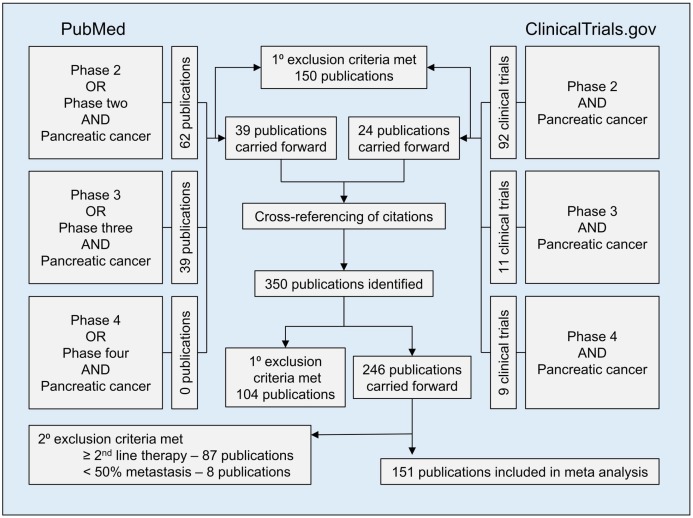
Flow diagram showing methods for inclusion and exclusion of studies in meta-analysis For the PubMed search, the ‘phase’ search term was limited to the title/abstract, whereas ‘pancreatic cancer’ was not restricted by search location. For the www.clinicaltrials.gov search, results were limited to completed interventional trials with results. Primary exclusion criteria included: 1) trials in which any patient had ampullary carcinoma, intraductal papillary mucinous neoplasm, hepatobiliary carcinoma, or any diagnosis other than PDAC; 2) publications not available in English; 3) trials which included any patient with resectable or borderline resectable disease; 4) trials evaluating either adjuvant or primarily radiation therapy; 5) sub-analyses of previously published clinical trials; and 6) meeting proceedings and abstracts without an associated published manuscript.

A total of 350 publications were obtained after cross-referencing articles identified through searches of PubMed and www.ClinicalTrials.gov. Of these, 104 were excluded based on primary exclusion criteria. Of the 246 publications carried forward, 87 clinical trials investigated second-line therapy or higher, and eight clinical trials enrolled less than 50% of study participants with metastatic disease, and were thus excluded. A total of 151 publications were included in the meta-analysis (Figure 1, List of clinical trials in Supplementary Materials).

Since 1986, 19,488 patients have participated in 151 published Phase Two (*N* = 121) and Three (*N* = 30) clinical trials (Table 1). No Phase Four clinical trials were identified. Sixty-eight percent of clinical trials allowed for inclusion of patients with either LAPC or mPDAC; however, 84% of all study participants had metastatic disease (Table 1). Clinical trials originated from sixteen different countries, 40% from the United States (*N* = 60). Clinical trials from other countries included France (*N* = 16), Italy (*N* = 13), Germany (*N* = 12) Greece (*N* = 8), Japan (*N* = 8), and the United Kingdom (*N* = 8) (Table 1).

**Table 1:** Characteristics of study participants and clinical trials

	Characteristic(s)	Number		Percent	
Study participants					
	Disease stage				
	Metastatic	16,380			84.1%
	Locally advanced	3,058			15.7%
	Unknown	50			0.3%
	Gender				
	Male	10,880	55.8%		
	Female	8,236	42.3%		
	Unknown	372	1.9%		
	Age (weighted median, years)	62.5			
Clinical trials					
	Inclusion criteria				
	Locally advanced and metastatic	102			67.5%
	Metastatic only	49			32.5%
	Trial phase				
	Phase two	121			80.1%
	Phase three	30			19.9%
	Country of origin				
	United States	60			39.7%
	France	16			10.6%
	Italy	13			8.6%
	Germany	12			7.9%
	Greece	8			5.3%
	Japan	8			5.3%
	United Kingdom	8			5.3%
	Austria	5			3.3%
	Canada	5			3.3%
	Netherlands	4			2.6%
	Belgium	3			2.0%
	Spain	3			2.0%
	Switzerland	3			2.0%
	Other	3			2.0%

### Efficacy by treatment regimen

**Figure 2:**
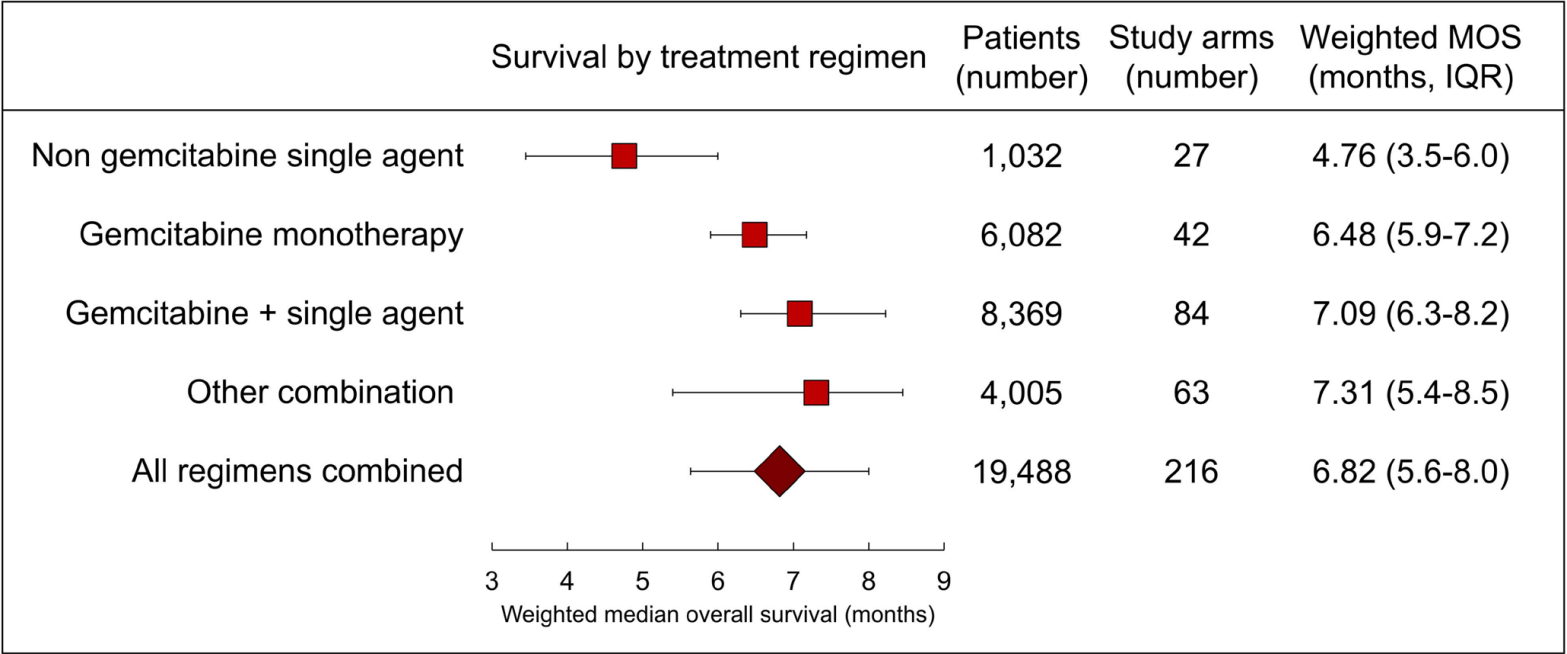
Forest plot of treatment efficacy Weighted MOS and interquartile range (IQR) for all treatment regimens are plotted.

Median wMOS was numerically higher in combination therapy (7.31 months, IQR 5.4 to 8.5) compared to non-gemcitabine, single-agent therapy (4.76 months, IQR 3.5 to 6.0), gemcitabine monotherapy (6.48 months, IQR 5.9 to 7.2), and gemcitabine plus single-agent therapy (7.09 months, IQR 6.3 to 8.2) (Figure 2). For all drugs or regimens used in two or more study arms, FOLFIRINOX was the most efficacious combination regimen (wMOS 10.9 months) [6, 7], S-1 was the most efficacious single agent regimen (wMOS 8.0 months) [8, 9], and nab-paclitaxel was the most efficacious agent when administered in addition to gemcitabine (wMOS 9.0 months) [10, 11] (Table 2). The highest MOS reported in any single study arm was 12.5 months in a single-arm, Phase Two clinical trial of 33 patients (all of whom had metastatic disease) who were treated with gemcitabine and S-1 [12] (Table 3).

**Table 2:** Drug(s) used in more than one study arm

Regimen	Drug(s)	Study arms (number)	Weighted MOS (months)
Gemcitabine only	Gemcitabine	42	6.5
	5-FU	7	4.3
Single agent	S-1	2	8.0
[non-gemcitabine]	Docetaxel	2	5.2
	Cisplatin	11	7.4
	5-FU	9	7.1
	Capecitabine	7	7.9
	Erlotinib	7	6.5
	Docetaxel	6	7.3
Gemcitabine plus	Oxaliplatin	5	7.1
single agent	Irinotecan	4	6.6
	Ganitumab	3	7.2
	Sorafenib	3	6.8
	Nab-paclitaxel	2	9.0
	Axitinib	2	8.2
	Cetuximab	2	6.4
	Bevacizumab	2	6.2
	Cisplatin, 5-FU, gemcitabine	4	7.8
	Uracil-tegafur	4	7.5
	5-FU, IFN-alpha	4	5.1
	Docetaxel, irinotecan	3	8.0
	Cisplatin, 5-FU	3	5.7
Other combination	Epirubicin, 5-FU	3	5.4
	FOLFIRINOX	2	10.9
	FOLFOX	2	9.7
	Capecitabine, gemcitabine, GV1001	2	7.7
	Capecitabine, erlotinib, gemcitabine	2	6.5
	Cisplatin, ARA-c, caffeine	2	5.5

**Table 3:** Drug(s) with the highest weighted MOS, for all study arms

Regimen	Drug(s)	Study arms (number)	Weighted MOS (months)
Gemcitabine only	Gemcitabine	42	6.5
	PHY906	1	8.2
	S-1	2	8.0
	Goserelin	1	7.5
Single agent	LY231514	1	6.5
[non-gemcitabine]	D-Trp-6-LH-RH	1	6.0
	Glufosfamide	1	5.3
	Irinotecan	1	5.2
	Docetaxel	2	5.2
	Buserelin	1	5.0
	S-1	1	12.5
	Nab-paclitaxel	2	9.0
	3-AP	1	9.0
	Trametinib	1	8.4
Gemcitabine plus	Axitinib	2	8.2
single agent	Tigatuzumab	1	8.2
	Capecitabine	7	7.9
	Conatumumab	1	7.5
	Carboplatin	1	7.4
	Cisplatin	11	7.4
	FOLFIRI	1	12.1
	Gemcitabine, FOLFIRI	1	11.0
	FOLFIRINOX	2	10.9
	5-FU, mitomycin, streptozotocin	1	10.0
Other combination	Bevacizumab, capecitabine, gemcitabine	1	9.8
	FOLFOX	2	9.7
	Cisplatin, epirubicin, 5-FU, gemcitabine	1	9.5
	Cisplatin, S-1	1	9.0
	Docetaxel, GCSF	1	8.3
	Capecitabine, oxaliplatin	1	8.1

### Trends in treatment efficacy

**Figure 3:**
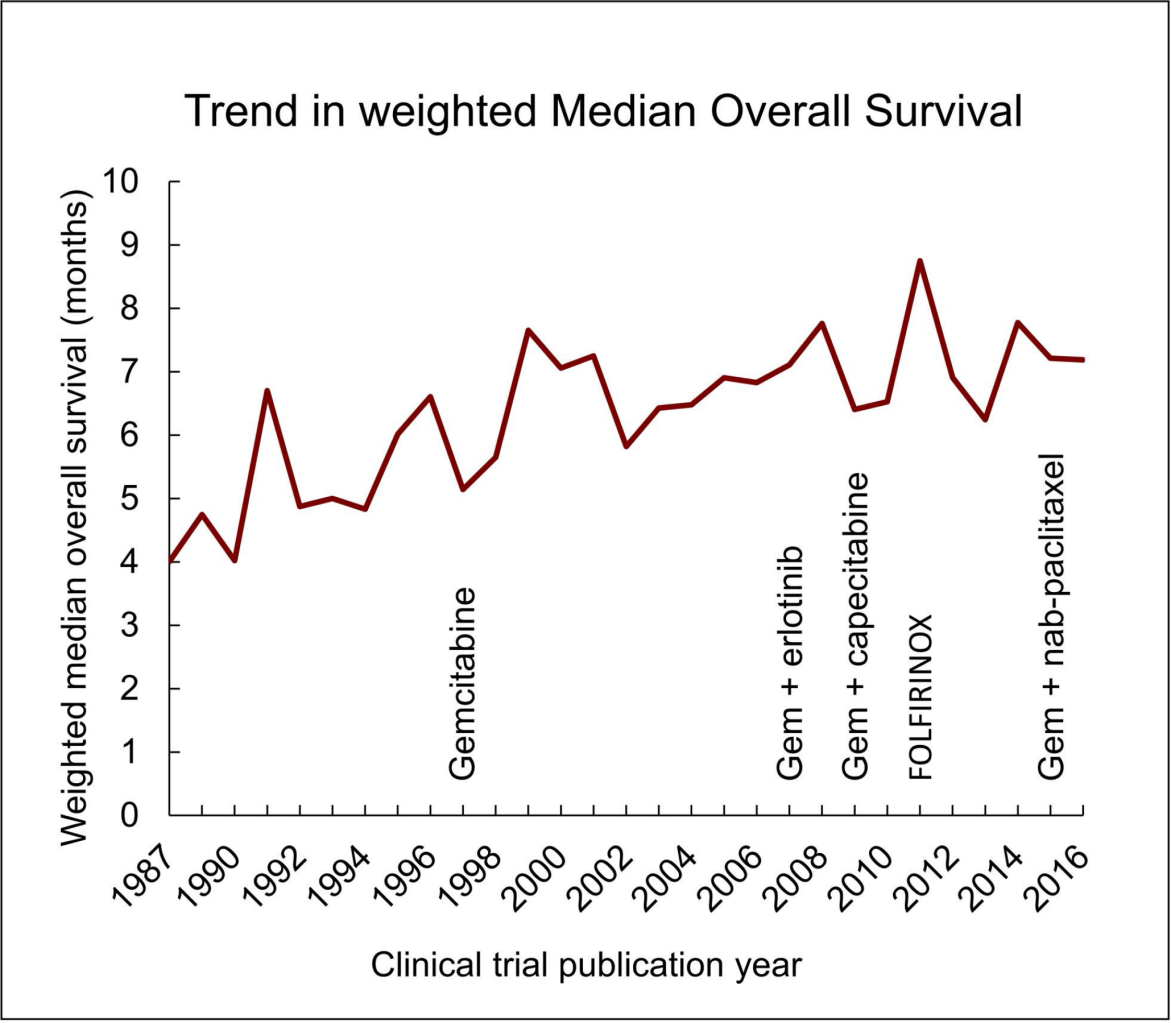
Trends in weighted MOS Drug regimens are listed in order of publication in large phase three clinical trials [1, 11, 28, 29].

Weighted MOS increased from 4.74 to 7.75 months from 1986 to 2016, an absolute and relative increase of 3.01 months and 64%, respectively (Figure 3 and Supplementary Table 1). Extrapolation of this trend demonstrates that a wMOS of 12 months will not be achieved until the year 2059 (Supplementary Table 1). However, analysis of wMOS trends since 1998 (shortly after the introduction of gemcitabine) estimates that a 12-month wMOS will not be achieved until the year 2127 (Supplementary Table 2). No significant difference was found between the number of study arms reporting biased-high (*N* = 6) versus biased-low (*N* = 10) (*P* = 0.31) MOS (Supplementary Figure 1).

## DISCUSSION

Herein, we describe our performance of a meta-analysis of survival in clinical trials of primarily mPDAC. The 151 clinical trials included in this meta-analysis capture the vast majority of clinical trials that investigated first-line systemic chemotherapy in mPDAC. Our study is unique in many aspects. This meta-analysis is extensive, including as noted, 151 clinical trials and 19,488 participants over a period of thirty years, which is by far the largest selection analyzed to date. Ours is also the first study to quantify the overall progress of clinical trials in patients with mPDAC, and in having done so, we demonstrate that, accounting for the data from all clinical trials to date, patient survival has only increased by three months in thirty years.

The current lifetime risk of any one person developing PDAC is 1.6%, and by 2025 PDAC is expected to surpass colon cancer to become the second-most common cause of cancer-related mortality [4, 13]. Over the same time, PDAC will likely surpass breast and colon cancer to become the second-most common cause of years of life lost to cancer [2, 13]. If we fail to effectively screen or treat PDAC, projected increases in incidence will lead to nearly parallel increases in mortality.

Few regimens have demonstrated meaningful superiority over gemcitabine since its discovery by Burris and colleagues in 1997 [1]. In 2011, Conroy and colleagues found that FOLFIRINOX provided an additional 4.3 months of survival benefit over gemcitabine in a randomized phase three trial, arguably the biggest therapeutic advancement for patients with mPDAC to date [7]. However, the routine clinical use of this regimen is limited by its toxicity and not all patients are suitable candidates. More robust data are needed on its effectiveness outside of a clinical trial. Our analysis further demonstrates that for patients with mPDAC, gemcitabine is associated with a MOS of 6.5 months, which is not markedly different than other treatment regimens, including single agent, gemcitabine and single agent, and other combination therapy. It is also important to note that our analysis was not restricted to only cytotoxic therapies, but included biologic and immunotherapy drugs, as well as other classes of treatment, further underscoring the difficulty of identifying effective treatment regimens in PDAC.

Several mechanisms contribute to treatment failure in PDAC including cancer stem cell resistance (CSC), stromal-related tumor effects, and immune system evasion. In 2007, Li and colleagues first identified pancreatic CSCs which expressed CD44/CD24/ESA+ antigen markers [14]. Others markers of stemness have since been identified and include CD133, ALDH, c-Met, DCLK1, and CD166 [15–19]. These populations of cells are often resistant to traditional chemotherapeutic agents and may contribute to treatment failure. Pancreatic stroma also promotes tumor growth and inhibits the delivery of therapeutic agents through modulation of the tumor microenvironment and elevation of interstitial pressure, respectively [20, 21]. Prior work by Provenzano and colleagues demonstrated that hyaluronan reduced the interstitial pressure in murine tumors, resulting in microvascular expansion and increased survival when given in addition to gemcitabine [21]. Given the recent success of immunotherapy in melanoma and other cancers, efforts are underway to investigate its utility in patients with PDAC; however, comparable survival benefits have not yet been observed. This may be the result of PDAC having a lower mutational rate compared to other cancers [22–24]. Additionally, Tregs, Macrophages, and myeloid-derived suppressive cells within the tumor suppress the immune response, further promoting cancer cell survival [22, 24]. Several trials are underway investigating the potential benefits of immunotherapy in patients with advanced PDAC (Supplementary Table 3). It will likely be several more years before a consensus is reached regarding the utility of immunotherapy in PDAC.

This study focuses on progress made in the management of mPDAC, which patients accounted for 84% of our study population. Thus, the patient population of this study excludes patients who are eligible for curative approaches. As such, our findings may not reflect the advances made in surgical and radiation treatment. Nevertheless, while the results of our analyses demonstrate stagnation in the overall survival for patients with mPDAC over the past thirty years, it might also be argued that no progress of any kind had been seen prior to 1986, further strengthening our findings. Finally, these findings are of significant value given that 52% of patients have metastatic PDAC at initial diagnosis [25].

This study has a few limitations. Although our methods were comprehensive, human error may have resulted in unintentional inclusion or exclusion of trials. To minimize this risk, multiple collaborators were involved in data collection. In addition, we considered the fact that progression-free survival and time to progression vary in definition and were not uniformly reported in all studies, and therefore elected not to report on those findings. Several trials were multinational in design, which made comparisons of country of origin difficult; in consideration of this, we used the address of the corresponding author as the “country of origin” (we still find this to be a useful way to estimate global distribution of clinical trials). Considering that a true estimate of study error was not consistently reported in all included clinical trials, confidence intervals were not appropriate to compare treatment efficacy among all four groups (non-gemcitabine single agent, gemcitabine monotherapy, gemcitabine plus single agent, and other combinations). Thus, we reported the median wMOS and interquartile range for all data, which we believe is more appropriate for this analysis. For similar reasons, we were unable to construct a funnel plot with true 95% limits; rather, the limits represent a heuristically derived boundary to identify bias in one direction or another, for which none existed (Supplementary Figure 1).

This data, in combination with other survival statistics, demonstrate that previous research efforts focusing on treatment of PDAC have not led to meaningful gains in survival of metastatic PDAC. In light of the increasing incidence and mortality combined with stagnant survival rates, a new approach to improving survival in metastatic PDAC may be warranted, including a stronger emphasis on the identification of novel targets for early detection, screening, and intervention.

## MATERIALS AND METHODS

### Search strategy and selection criteria

The search for clinical trials in mPDAC was conducted using both www.ClinicalTrials.gov and PubMed, in accordance with PRISMA guidelines [26]. A search of https://clinicaltrials.gov/ for Phases Two through Four registered, closed interventional clinical trials, using the search term “pancreatic cancer,” was performed on January 5th, 2017. The PubMed search was conducted on February 7th, 2017 for publications containing either “Phase 2” or “Phase Two” in the title or abstract, and “pancreatic cancer” elsewhere in the text. Similar searches were performed for Phases Three and Four clinical trials. Extensive cross-referencing of citations was performed to capture as many relevant clinical trials as possible (Figure 1). To ensure accuracy of clinical trials, all data were collected and reviewed by co-authors. Discrepancies were discussed until a consensus was reached, at which point appropriate changes were made when necessary.

Inclusion criteria were: 1) Phase Two, Three, or Four clinical trials that included patients with mPDAC; 2) trials published during or after 1986; 3) trials investigating the benefit of first-line systemic chemotherapy; and 4) trials reporting MOS rate(s). Primary exclusion criteria included: 1) trials in which any patient had ampullary carcinoma, intraductal papillary mucinous neoplasm, hepatobiliary carcinoma, or any diagnosis other than PDAC; 2) publications not available in English; 3) trials that included any patient with resectable or borderline resectable disease; 4) trials evaluating either adjuvant or primarily radiation therapy; 5) sub-analyses of previously published clinical trials; and 6) meeting proceedings and abstracts without an associated published manuscript. Secondary exclusion criteria included: 1) trials in which fewer than 50% of study patients had metastatic disease, with the majority having locally advanced (LAPC) disease; and 2) trials investigating lines of therapy other than first-line therapy.

### Data analysis

The sample mean, median, standard deviation, and interquartile range were calculated. Weighted MOS was calculated using the MOS rate reported for each study arm, weighted by the number of study participants in each respective study arm where *a* = number of studies, *b* = number of drug combinations, and *c* = number of study arms (Equation 1) [27]. Weighted median age was calculated similarly. Trend lines were calculated using linear regression modeling and used to determine the change in wMOS over time, including future projections. Assessment for study bias was performed by creation of a funnel plot (R statistical software version 3.3.1) using the MOS and number of study participants reported for each respective study arm (Equation 2). The published address of the corresponding author was used to designate the “country of origin” of individual trials. “Single-agent therapy” was defined as treatment with a single drug other than gemcitabine. “Other combination” therapy included any combination of three or more drugs, or two drugs so long as neither was gemcitabine.

$$wMOS=\sum_{i=1}^{a}\sum_{j=1}^{b}\sum_{k=1}^{c}MOS_{ijk}\frac{n_{ijk}}{n_{ij.}}$$ *Equation*1

$$Boundary=\exp\left(\ln\left(wMOS\right)\pm 1.96\frac{6*sd\left(\ln\left(MOS\right)\right)}{\sqrt{n_{arm}}}\right)$$ *Equation*2

### Role of the funding source

The funding source of the study had no role in study design, data collection, data analysis, data interpretation, or writing the report. The corresponding author had full access to all data in the study and has final responsibility for the decision to submit for publication.

## SUPPLEMENTARY MATERIALS FIGURE AND TABLES


