# Supplementary material for: Advanced pancreatic cancer: a meta-analysis of clinical trials over thirty years
Source: Oncotarget. 2018 Apr 10;9(27):19396–405. doi: 10.18632/oncotarget.25036 (PMC5922405; doi:10.18632/oncotarget.25036)
Supplement: Supplementary file 1 [file oncotarget-09-19396-s001.pdf]

Advanced pancreatic cancer: a meta-analysis of clinical trials over thirty years

SUPPLEMENTARY MATERIALS

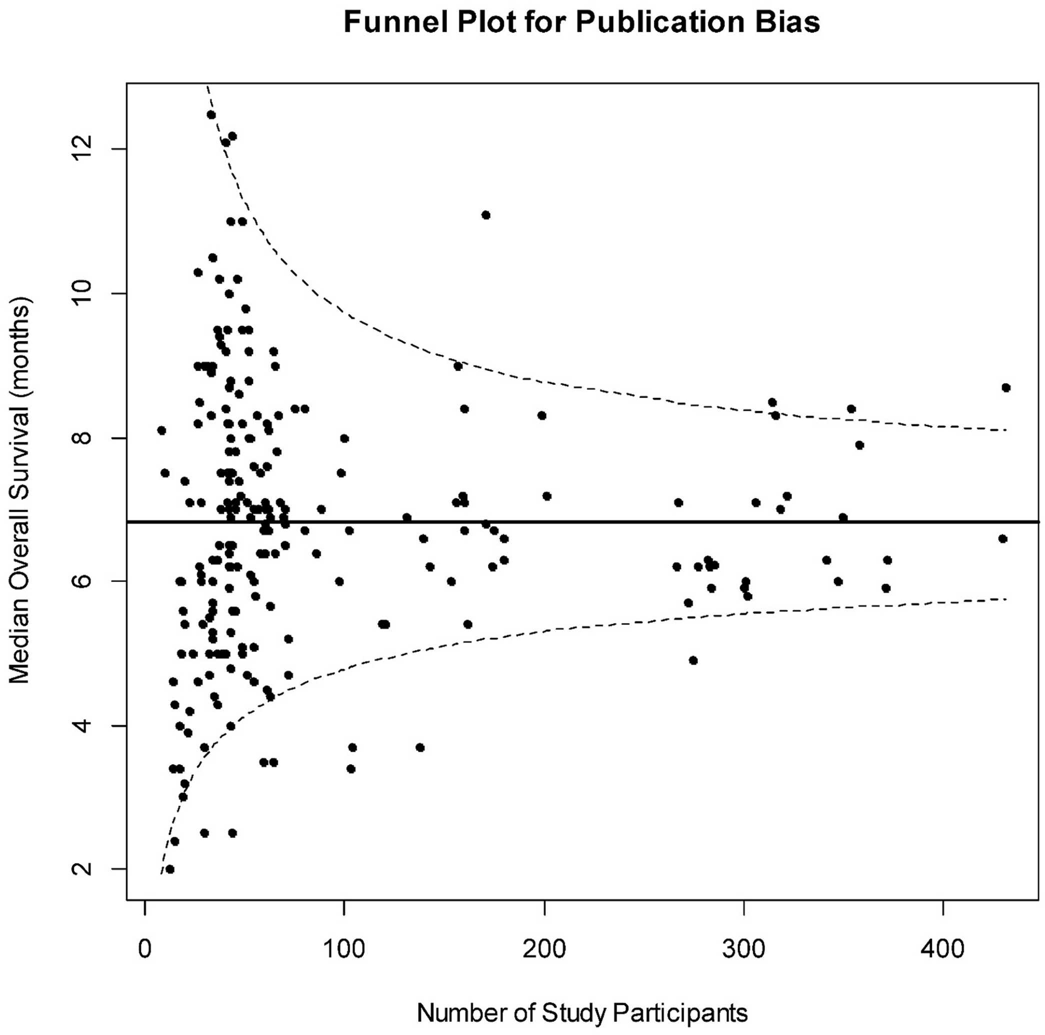

Supplementary Figure 1: Funnel plot of survival and MOS in each study arm.

Supplementary Table 1: Change in wMOS since 1986

Year	Weighted MOS (months)
1986	4.74
2016	7.75
Absolute increase	3.01
Relative increase (%)	64%

Estimated year when wMOS surpasses 12 months = 2059.

Supplementary Table 2: Change in wMOS since 1998

Year	Weighted MOS (months)
1998	6.56
2016	7.33
Absolute increase	0.76
Relative increase (%)	12%

Estimated year when wMOS surpasses 12 months = 2127.

Supplementary Table 3: Select clinical trials investigating immunotherapy in advanced PDAC

Trial ID	Brief Title	Treatment(s)
**Checkpoint Inhibitor Based Approaches**		
NCT03190265	Study of CRS-207, Nivolumab, and Ipilimumab With or Without GVAX Pancreas Vaccine (With Cy) in Patients With Pancreatic Cancer	1. Nivolumab<br>2. Ipilimumab<br>3. GVAX<br>4. CRS-207<br>5. Cyclophosphamide
NCT03193190	A Study of Multiple Immunotherapy-Based Treatment Combinations in Participants With Metastatic Pancreatic Ductal Adenocarcinoma (Morpheus-Pancreatic Cancer)	1. Atezolizumab<br>2. Cobimetinib<br>3. PEGPH20<br>4. BL-8040<br>5. Nab-paclitaxel<br>6. Gemcitabine<br>7. mFOLFOX6
NCT03168139	Olaptesed (NOX-A12) Alone and in Combination With Pembrolizumab in Colorectal and Pancreatic Cancer (Keynote-559)	1. Olaptesed pegol<br>2. Pembrolizumab
NCT03098550	A Study to Test the Safety and Effectiveness of Nivolumab Combined With Daratumumab in Patients With Pancreatic, Non-Small Cell Lung or Triple Negative Breast Cancers, That Have Advanced or Have Spread	1. Nivolumab<br>2. Daratumumab
NCT02734160	A Study of Galunisertib (LY2157299) and Durvalumab (MEDI4736) in Participants With Metastatic Pancreatic Cancer	1. Galunisertib<br>2. Durvalumab
NCT03006302	Epacadostat, Pembrolizumab, and CRS-207, With or Without CY/GVAX Pancreas in Patients With Metastatic Pancreas Cancer	1. Epacadostat<br>2. Pembrolizumab<br>3. CRS-207<br>4. GVAX<br>5. Cyclophosphamide
NCT00836407	Ipilimumab +/- Vaccine Therapy in Treating Patients With Locally Advanced, Unresectable or Metastatic Pancreatic Cancer	1. Ipilimumab<br>2. Pancreatic cancer vaccine (5E8 cells)
**Cell Based Immune Therapy Approaches**		
NCT03323944	CAR T Cell Immunotherapy for Pancreatic Cancer	1. huCART-meso cells
NCT03008304	High-activity Natural Killer Immunotherapy for Small Metastases of Pancreatic Cancer	1. High-activity NK cells
NCT01174121	Immunotherapy Using Tumor Infiltrating Lymphocytes for Patients With Metastatic Cancer	1. Young TIL<br>2. Aldesleukin<br>3. Fludarabine<br>4. Pembrolizumab<br>5. Cyclophosphamide
**Other Immune Approaches**		
NCT03086642	Study of Talimogene Laherparepvec (T-VEC) in Pancreatic Cancer	1. Talimogene Iaherparepvec
NCT01417000	Safety and Efficacy of Combination Listeria/GVAX Immunotherapy in Pancreatic Cancer	1. GVAX<br>2. CRS-207<br>3. Cyclophosphamide

REFERENCES (CLINICAL TRIALS INCLUDED IN META-ANALYSIS)

1. Abou-Alfa GK, Letourneau R, Harker G, Modiano M, Hurwitz H, Tchekmedyian NS, Feit K, Ackerman J, De Jager RL, Eckhardt SG, O'reilly EM. Randomized phase III study of exatecan and gemcitabine compared with gemcitabine alone in untreated advanced pancreatic cancer. J Clin Oncol. 2006; 24:4441–7.
2. Alberts SR, Foster NR, Morton RF, Kugler J, Schaefer P, Wiesenfeld M, Fitch TR, Steen P, Kim GP, Gill S. PS-341 and gemcitabine in patients with metastatic pancreatic adenocarcinoma: a North Central Cancer Treatment Group (NCCTG) randomized phase II study. Ann Oncol. 2005; 16:1654–61.
3. Alberts SR, Townley PM, Goldberg RM, Cha SS, Sargent DJ, Moore DF, Krook JE, Pitot HC, Fitch TR, Wiesenfeld M, Mailliard JA. Gemcitabine and oxaliplatin for metastatic pancreatic adenocarcinoma: a North Central Cancer Treatment Group phase II study. Ann Oncol. 2003; 14:580–5.
4. Andre T, Lotz JP, Bouleuc C, Azzouzi K, Houry S, Hannoun L, See J, Esteso A, Avenin D, Izrael V. Phase II trial of 5-fluorouracil, leucovorin and cisplatin for treatment of advanced pancreatic adenocarcinoma. Ann Oncol. 1996; 7:173–8.
5. Andren-Sandberg A. Treatment with an LHRH analogue in patients with advanced pancreatic cancer. A preliminary report. Acta Chir Scand. 1990; 156:549–51.
6. Androulakis N, Kourousis C, Dimopoulos MA, Samelis G, Kakolyris S, Tsavaris N, Genatas K, Aravantinos G, Papadimitriou C, Karabekios S, Stathopoulos GP, Georgoulias V. Treatment of pancreatic cancer with docetaxel and granulocyte colony-stimulating factor: a multicenter phase II study. J Clin Oncol. 1999; 17:1779.
7. Aranda E, Manzano JL, Rivera F, Galán M, Valladares-Ayerbes M, Pericay C, Safont MJ, Mendez MJ, Irigoyen A, Arrivi A, Sastre J, Diaz-Rubio E. Phase II open-label study of erlotinib in combination with gemcitabine in unresectable and/or metastatic adenocarcinoma of the pancreas: relationship between skin rash and survival (Pantar study). Ann Oncol. 2011; 23:1919–25.
8. Auerbach M, Wampler GL, Lokich JJ, Fryer D, Fryer JG, Ahlgren JD. Treatment of advanced pancreatic carcinoma with a combination of protracted infusional 5-fluorouracil and weekly carboplatin: a Mid-Atlantic Oncology Program Study. Ann Oncol. 1997; 8:439–44.
9. Berlin JD, Adak S, Vaughn DJ, Flinker D, Blaszkowsky L, Harris JE, Benson III AA. A phase II study of gemcitabine and 5-fluorouracil in metastatic pancreatic cancer: an Eastern Cooperative Oncology Group Study (E3296). Oncology. 2000; 58:215–8.
10. Berlin JD, Catalano P, Thomas JP, Kugler JW, Haller DG, Benson III AB. Phase III study of gemcitabine in combination with fluorouracil versus gemcitabine alone in patients with advanced pancreatic carcinoma: Eastern Cooperative Oncology Group Trial E2297. J Clin Oncol. 2002; 20:3270–5.
11. Boeck S, Hoehler T, Seipelt G, Mahlberg R, Wein A, Hochhaus A, Boeck HP, Schmid B, Kettner E, Stauch M, Lordick F, Ko Y, Geissler M, et al. Capecitabine plus oxaliplatin (CapOx) versus capecitabine plus gemcitabine (CapGem) versus gemcitabine plus oxaliplatin (mGemOx): final results of a multicenter randomized phase II trial in advanced pancreatic cancer. Ann Oncol. 2007; 19:340–7.
12. Bolli E, Saccomanno S, Mondini G, Aschele C, Guglielmi A, Ligas B, Connio M, Mori A, Rosso R, Sobrero A, Tixi L, Pugliese V, GLISP (Gruppo Ligure Studio Pancreas). 5-Fluorouracil plus 5-methyltetrahydrofolate in advanced pancreatic cancer. Cancer Chemother Pharmacol. 1995; 35:339–42.
13. Bramhall SR, Schulz J, Nemunaitis J, Brown PD, Baillet M, Buckels JA. A double-blind placebo-controlled, randomised study comparing gemcitabine and marimastat with gemcitabine and placebo as first line therapy in patients with advanced pancreatic cancer. Br J Cancer. 2002; 87:161.
14. Briasoulis E, Pavlidis N, Terret C, Bauer J, Fiedler W, Schöffski P, Raoul JL, Hess D, Selvais R, Lacombe D, Bachmann P, Fumoleau P. Glufosfamide administered using a 1-hour infusion given as first-line treatment for advanced pancreatic cancer. A phase II trial of the EORTC-new drug development group. Eur J Cancer. 2003; 39:2334–40.
15. Burris 3rd HA, Moore MJ, Andersen J, Green MR, Rothenberg ML, Modiano MR, Cripps MC, Portenoy RK, Storniolo AM, Tarassoff P, Nelson R, Dorr FA, Stephens CD, et al. Improvements in survival and clinical benefit with gemcitabine as first-line therapy for patients with advanced pancreas cancer: a randomized trial. J Clin Oncol. 1997; 15:2403–13.
16. Burtness B, Powell M, Catalano P, Berlin J, Liles DK, Chapman AE, Mitchell E, Benson AB. Randomized phase II trial of irinotecan/docetaxel or irinotecan/docetaxel plus cetuximab for metastatic pancreatic cancer: an eastern cooperative oncology group study. Am J Clin Oncol. 2016; 39:340.
17. Burtness B, Thomas L, Sipples R, McGurk M, Salikooti S, Christoforou M, Mirto G, Salem R, Sosa J, Kloss R, Rahman Z, Chung G, Lacy J, et al. Phase II trial of weekly docetaxel/irinotecan combination in advanced pancreatic cancer. Cancer J. 2007; 13:257–62.
18. Canobbio L, Boccardo F, Cannata D, Gallotti P, Epis R. Treatment of advanced pancreatic carcinoma with the somatostatin analogue BIM 23014. Preliminary results of a pilot study. Cancer. 1992; 69:648–50.
19. Carmichael J, Fink U, Russell RC, Spittle MF, Harris AL, Spiessi G, Blatter J. Phase II study of gemcitabine in patients with advanced pancreatic cancer. Br J Cancer. 1996; 73:101–5.

20. Cascinu S, Berardi R, Labianca R, Siena S, Falcone A, Aitini E, Barni S, Di Costanzo F, Dapretto E, Tonini G, Pierantoni C, Artale S, Rota S, et al. Cetuximab plus gemcitabine and cisplatin compared with gemcitabine and cisplatin alone in patients with advanced pancreatic cancer: a randomised, multicentre, phase II trial. Lancet Oncol. 2008; 9:39–44.
21. Cascinu S, Berardi R, Sobrero A, Bidoli P, Labianca R, Siena S, Ferrari D, Barni S, Aitini E, Zagonel V, Caprioni F, Villa F, Mosconi S, et al. Sorafenib does not improve efficacy of chemotherapy in advanced pancreatic cancer: A GISCAD randomized phase II study. Dig Liver Dis. 2014; 46:182–6.
22. Cascinu S, Fedeli A, Fedeli SL, Catalano G. 5-Fluorouracil, leucovorin and interferon alpha 2b in advanced pancreatic cancer: a pilot study. Ann Oncol. 1993; 4:83–4.
23. Cascinu S, Frontini L, Labianca R, Catalano V, Barni S, Graiff C, Picone G, Farinati F, Zonato S, Pessi MA, Curti C, Catalano G. A combination of a fixed dose rate infusion of gemcitabine associated to a bolus 5-fluorouracil in advanced pancreatic cancer, a report from the Italian Group for the Study of Digestive Tract Cancer (GISCAD). Ann Oncol. 2000; 11:1309–11.
24. Cascinu S, Labianca R, Catalano V, Barni S, Ferraù F, Beretta GD, Frontini L, Foa P, Pancera G, Priolo D, Graziano F, Mare M, Catalano G. Weekly gemcitabine and cisplatin chemotherapy: a well-tolerated but ineffective chemotherapeutic regimen in advanced pancreatic cancer patients. A report from the Italian Group for the Study of Digestive Tract Cancer (GISCAD). Ann Oncol. 2003; 14:205–8.
25. Cascinu S, Silva RR, Barni S, Labianca R, Frontini L, Piazza E, Pancera G, Giordani P, Giuliodori L, Pessi MA, Fusco V, Luporini G, Cellerino R, et al. A combination of gemcitabine and 5-fluorouracil in advanced pancreatic cancer, a report from the Italian Group for the Study of Digestive Tract Cancer (GISCAD). Br J Cancer. 1999; 80:1595–8.
26. Casper ES, Green MR, Kelsen DP, Heelan RT, Brown TD, Flombaum CD, Trochanowski B, Tarassoff PG. Phase II trial of gemcitabine (2, 2′-difiuorodeoxycytidine) in patients with adenocarcinoma of the pancreas. Invest New Drugs. 1994; 12:29–34.
27. Catenacci DV, Junttila MR, Karrison T, Bahary N, Horiba MN, Nattam SR, Marsh R, Wallace J, Kozloff M, Rajdev L, Cohen D, Wade J, Sleckman B, et al. Randomized phase Ib/II study of gemcitabine plus placebo or vismodegib, a Hedgehog pathway inhibitor, in patients with metastatic pancreatic cancer. J Clin Oncol. 2015; 33:4284–92.
28. Chee CE, Krishnamurthi S, Nock CJ, Meropol NJ, Gibbons J, Fu P, Bokar J, Teston L, O'Brien T, Gudena V, Reese A, Bergman M, Saltzman J, et al. Phase II study of dasatinib (BMS-354825) in patients with metastatic adenocarcinoma of the pancreas. Oncologist. 2013; 18:1091–2.
29. Chen J, Röcken C, Nitsche B, Hosius C, Gschaidmeier H, Kahl S, Malfertheiner P, Ebert MP. The tyrosine kinase inhibitor imatinib fails to inhibit pancreatic cancer progression. Cancer Lett. 2006; 233:328–37.
30. Clayton AJ, Mansoor AW, Jones ET, Hawkins RE, Saunders MP, Swindell R, Valle JW. A phase II study of weekly cisplatin and gemcitabine in patients with advanced pancreatic cancer: is this a strategy still worth pursuing? Pancreas. 2006; 32:51–7.
31. Cohen SJ, Zalupski MM, Conkling P, Nugent F, Ma WW, Modiano M, Rolan P, Lee FC, Wong L, Hersh E. A Phase 2 Randomized, Double-Blind, Multicenter Trial of Imexon Plus Gemcitabine Versus Gemcitabine Plus Placebo in Patients With Metastatic Chemotherapy-naive Pancreatic Adenocarcinoma. Am J Clin Oncol. 2018; 41:230–5.
32. Colucci G, Giuliani F, Gebbia V, Biglietto M, Rabitti P, Uomo G, Cigolari S, Testa A, Maiello E, Lopez M. Gemcitabine alone or with cisplatin for the treatment of patients with locally advanced and/or metastatic pancreatic carcinoma. Cancer. 2002; 94:902–10.
33. Colucci G, Labianca R, Di Costanzo F, Gebbia V, Cartenì G, Massidda B, Dapretto E, Manzione L, Piazza E, Sannicolo M, Ciaparrone M, Cavanna L, Guiliani F, et al. Randomized phase III trial of gemcitabine plus cisplatin compared with single-agent gemcitabine as first-line treatment of patients with advanced pancreatic cancer: the GIP-1 study. J Clin Oncol. 2010; 28:1645–51.
34. Conroy T, Desseigne F, Ychou M, Bouché O, Guimbaud R, Bécouarn Y, Adenis A, Raoul JL, Gourgou-Bourgade S, de la Fouchardière C, Bennouna J, Bachet JB, Khemissa-Akouz F, et al. FOLFIRINOX versus gemcitabine for metastatic pancreatic cancer. N Engl J Med. 2011; 364:1817–25.
35. Conroy T, Paillot B, François E, Bugat R, Jacob JH, Stein U, Nasca S, Metges JP, Rixe O, Michel P, Magherini E, Hua A, Deplanque G. Irinotecan plus oxaliplatin and leucovorin-modulated fluorouracil in advanced pancreatic cancer—a Groupe Tumeurs Digestives of the Federation Nationale des Centres de Lutte Contre le Cancer study. J Clin Oncol. 2005; 23:1228–36.
36. Cullinan S, Moertel CG, Wieand HS, Schutt AJ, Krook JE, Foley JF, Norris BD, Kardinal CG, Tschetter LK, Barlow JF. A phase III trial on the therapy of advanced pancreatic carcinoma evaluations of the mallinson regimen and combined 5-fluorouracil, doxorubicin, and cisplatin. Cancer. 1990; 65:2207–12.
37. Cunningham D, Chau I, Stocken DD, Valle JW, Smith D, Steward W, Harper PG, Dunn J, Tudur-Smith C, West J, Falk S, Crellin A, Adab F, et al. Phase III randomized comparison of gemcitabine versus gemcitabine plus capecitabine in patients with advanced pancreatic cancer. J Clin Oncol. 2009; 27:5513–8.
38. Dahan L, Bonnetain F, Ychou M, Mitry E, Gasmi M, Raoul JL, Cattan S, Phelip JM, Hammel P, Chauffert B, Michel P,

Legoux JL, Rougier P, et al. Combination 5-fluorouracil, folinic acid and cisplatin (LV5FU2-CDDP) followed by gemcitabine or the reverse sequence in metastatic pancreatic cancer: final results of a randomised strategic phase III trial (FFCD 0301). Gut. 2010; 59:1527–34.

39. DeCaprio JA, Mayer RJ, Gonin R, Arbuck SG. Fluorouracil and high-dose leucovorin in previously untreated patients with advanced adenocarcinoma of the pancreas: results of a phase II trial. J Clin Oncol. 1991; 9:2128–33.

40. Di Costanzo F, Carlini P, Doni L, Massidda B, Mattioli R, Iop A, Barletta E, Moscetti L, Recchia F, Tralongo P, Gasperoni S. Gemcitabine with or without continuous infusion 5-FU in advanced pancreatic cancer: a randomised phase II trial of the Italian oncology group for clinical research (GOIRC). Br J Cancer. 2005; 93:185.

41. Dougherty JB, Kelsen D, Kemeny N, Magill G, Botet J, Niedzwiecki D. Advanced pancreatic cancer: a phase I–II trial of cisplatin, high-dose cytarabine, and caffeine. J Natl Cancer Inst. 1989; 81:1735–8.

42. Ducreux M, Mitry E, Ould-Kaci M, Boige V, Seitz JF, Bugat R, Breau JL, Bouche O, Etienne PL, Tigaud JM, Morvan F, Cvitkovic E, Rougier P. Randomized phase II study evaluating oxaliplatin alone, oxaliplatin combined with infusional 5-FU, and infusional 5-FU alone in advanced pancreatic carcinoma patients. Ann Oncol. 2004; 15:467–73.

43. Ducreux M, Rougier P, Pignon JP, Douillard JY, Seitz JF, Bugat R, Bosset JF, Merouche Y, Raoul JL, Ychou M, Adenis A, Berthault-Cvitkovic F, Luboinski M. A randomised trial comparing 5-FU with 5-FU plus cisplatin in advanced pancreatic carcinoma. Ann Oncol. 2002; 13:1185–91.

44. El-Khoueiry AB, Ramanathan RK, Yang DY, Zhang W, Shibata S, Wright JJ, Gandara D, Lenz HJ. A randomized phase II of gemcitabine and sorafenib versus sorafenib alone in patients with metastatic pancreatic cancer. Invest New Drugs. 2012; 30:1175–83.

45. El-Rayes BF, Zalupski MM, Shields AF, Vaishampayan U, Heilbrun LK, Jain V, Adsay V, Day J, Philip PA. Phase II study of gemcitabine, cisplatin, and infusional fluorouracil in advanced pancreatic cancer. J Clin Oncol. 2003; 21:2920–5.

46. Evans TR, Lofts FJ, Mansi JL, Glees JP, Dalgleish AG, Knight MJ. A phase II study of continuous-infusion 5-fluorouracil with cisplatin and epirubicin in inoperable pancreatic cancer. Br J Cancer. 1996; 73:1260.

47. Feliu J, Lopez Alvarez MP, Jaraiz MA, Constenla M, Vicent JM, Belon J, Lopez Gomez L, De Castro J, Dorta J, Gonzalez Baron M. Phase II trial of gemcitabine and UFT modulated by leucovorin in patients with advanced pancreatic carcinoma. Cancer. 2000; 89:1706–13.

48. Feliu J, Mel R, Borrega P, Lopez Gomez L, Escudero P, Dorta J, Castro J, Vazquez-Estevez SE, Bolanos M, Espinosa E, González Barón M. Phase II study of a fixed dose-rate infusion of gemcitabine associated with uracil/tegafur in advanced carcinoma of the pancreas. Ann Oncol. 2002; 13:1756–62.

49. Forero-Torres A, Infante JR, Waterhouse D, Wong L, Vickers S, Arrowsmith E, He AR, Hart L, Trent D, Wade J, Jin X, Wang Q, Austin T, et al. Phase 2, multicenter, open-label study of tigatuzumab (CS-1008), a humanized monoclonal antibody targeting death receptor 5, in combination with gemcitabine in chemotherapy-naive patients with unresectable or metastatic pancreatic cancer. Cancer Med. 2013; 2:925–32.

50. Frie H, Büchler M, Kruger M, Beger HG. Treatment of duct carcinoma of the pancreas with the LH-RH analogue buserelin. Pancreas. 1992; 7:516–21.

51. Fuchs CS, Azevedo S, Okusaka T, Van Laethem JL, Lipton LR, Riess H, Szczylik C, Moore MJ, Peeters M, Bodoky G, Ikeda M, Melichar B, Nemecek R, et al. A phase 3 randomized, double-blind, placebo-controlled trial of ganitumab or placebo in combination with gemcitabine as first-line therapy for metastatic adenocarcinoma of the pancreas: the GAMMA trial. Ann Oncol. 2015; 26:921–7.

52. Goldstein D, El-Maraghi RH, Hammel P, Heinemann V, Kunzmann V, Sastre J, Scheithauer W, Siena S, Tabernero J, Teixeira L, Tortora G, Van Laethem JL, Young R, et al. nab-Paclitaxel plus gemcitabine for metastatic pancreatic cancer: long-term survival from a phase III trial. J Natl Cancer Inst. 2015; 107:413.

53. Gonçalves A, Gilabert M, François E, Dahan L, Perrier H, Lamy R, Re D, Largillier R, Gasmi M, Tchiknavorian X, Esterni B, Genre D, Moureau-Zabotto L, et. al. BAYPAN study: a double-blind phase III randomized trial comparing gemcitabine plus sorafenib and gemcitabine plus placebo in patients with advanced pancreatic cancer. Ann Oncol. 2012; 23:2799–805.

54. Gonzalez-Barcena D, Ibarra-Olmos MA, Garcia-Carrasco F, Gutierrez-Samperio C, Comaru-Schally AM, Schally AV. Influence of D-Trp-6-LH-RH on the survival time in patients with advanced pancreatic cancer. Biomed Pharmacother. 1989; 43:313–7.

55. Gastrointestinal Tumor Study Group. Ifosfamide is an inactive substance in the treatment of pancreatic carcinoma. Cancer. 1989; 64:2010–3.

56. Halford S, Yip D, Karapetis CS, Strickland AH, Steger A, Khawaja HT, Harper PG. A phase II study evaluating the tolerability and efficacy of CAELYX (liposomal doxorubicin, Doxil) in the treatment of unresectable pancreatic carcinoma. Ann Oncol. 2001; 12:1399–402.

57. Heinemann V, Quietzsch D, Gieseler F, Gonnermann M, Schönekäs H, Rost A, Neuhaus H, Haag C, Clemens M, Heinrich B, Vehling-Kaiser U, Fuchs M, Fleckenstein D, et al. Randomized phase III trial of gemcitabine plus cisplatin compared with gemcitabine alone in advanced pancreatic cancer. J Clin Oncol. 2006; 24:3946–52.

58. Heinemann V, Vehling-Kaiser U, Waldschmidt D, Kettner E, Märten A, Winkelmann C, Klein S, Kojouharoff G, Gauler TC, von Weikersthal LF, Clemens MR, Geissler M, Greten TF, et al. Gemcitabine plus erlotinib followed by capecitabine versus capecitabine plus erlotinib followed

by gemcitabine in advanced pancreatic cancer: final results of a randomised phase 3 trial of the 'Arbeitsgemeinschaft Internistische Onkologie'(AIO-PK0104). Gut. 2013; 62:751–9.

59. Heinemann V, Wilke H, Mergenthaler HG, Clemens M, König H, Illiger HJ, Arning M, Schalhorn A, Possinger K, Fink U. Gemcitabine and cisplatin in the treatment of advanced or metastatic pancreatic cancer. Ann Oncol. 2000; 11:1399–403.

60. Herrmann R, Bodoky G, Ruhstaller T, Glimelius B, Bajetta E, Schuller J, Saletti P, Bauer J, Figer A, Pestalozzi B, Köhne CH, Mingrone W, Stemmer SM, et al. Gemcitabine plus capecitabine compared with gemcitabine alone in advanced pancreatic cancer: a randomized, multicenter, phase III trial of the Swiss Group for Clinical Cancer Research and the Central European Cooperative Oncology Group. J Clin Oncol. 2007; 25:2212–7.

61. Hess V, Pratsch S, Potthast S, Lee L, Winterhalder R, Widmer L, Cescato C, Lohri A, Jost L, Stillhart P, Pestalozzi B, Herrmann R. Combining gemcitabine, oxaliplatin and capecitabine (GEMOXEL) for patients with advanced pancreatic carcinoma (APC): a phase I/II trial. Ann Oncol. 2010; 21:2390–5.

62. Hess V, Salzberg M, Borner M, Morant R, Roth AD, Ludwig C, Herrmann R. Combining capecitabine and gemcitabine in patients with advanced pancreatic carcinoma: a phase I/II trial. J Clin Oncol. 2003; 21:66–8.

63. Hidalgo M, Castellano D, Paz-Ares L, Gravalos C, Diaz-Puente M, Hitt R, Alonso S, Cortes-Funes H. Phase I–II study of gemcitabine and fluorouracil as a continuous infusion in patients with pancreatic cancer. J Clin Oncol. 1999; 17:585–92.

64. Ina S, Tani M, Kawai M, Hirono S, Miyazawa M, Nishioka R, Fujita Y, Yamaue H. Phase 2 trial of oral S-1 combined with low-dose cisplatin for unresectable advanced pancreatic cancer. Anticancer Res. 2008; 28:2373–7.

65. Infante J, Arkenau HT, Bendell J, Rubin M, Waterhouse D, Jones G, Spigel D, Lane C, Hainsworth J, Burris, III H. Lenalidomide in combination with gemcitabine as first-line treatment for patients with metastatic carcinoma of the pancreas: a Sarah Cannon Research Institute phase II trial. Cancer Biol Ther. 2013; 14:340–6.

66. Infante JR, Somer BG, Park JO, Li CP, Scheulen ME, Kasubhai SM, Oh DY, Liu Y, Redhu S, Steplewski K, Le N. A randomised, double-blind, placebo-controlled trial of trametinib, an oral MEK inhibitor, in combination with gemcitabine for patients with untreated metastatic adenocarcinoma of the pancreas. Eur J Cancer. 2014; 50:2072–81.

67. Jacobs AD, Otero H, Picozzi Jr VJ, Aboulafia DM. Gemcitabine combined with docetaxel for the treatment of unresectable pancreatic carcinoma. Cancer Invest. 2004; 22:505–14.

68. Javle M, Yu J, Garrett C, Pande A, Kuvshinoff B, Litwin A, Phelan III J, Gibbs J, Iyer R. Bevacizumab combined with gemcitabine and capecitabine for advanced pancreatic cancer: a phase II study. Br J Cancer. 2009; 100:1842–5.

69. Kelsen D, Hudis C, Niedzwiecki D, Dougherty J, Casper E, Botet J, Vinciguerra V, Rosenbluth R. A phase III comparison trial of streptozotocin, mitomycin, and 5-fluorouracil with cisplatin, cytosine arabinoside, and caffeine in patients with advanced pancreatic carcinoma. Cancer. 1991; 68:965–9.

70. Kindler HL, Friberg G, Singh DA, Locker G, Nattam S, Kozloff M, Taber DA, Karrison T, Dachman A, Stadler WM, Vokes EE. Phase II trial of bevacizumab plus gemcitabine in patients with advanced pancreatic cancer. J Clin Oncol. 2005; 23:8033–40.

71. Kindler HL, Ioka T, Richel DJ, Bennouna J, Létourneau R, Okusaka T, Funakoshi A, Furuse J, Park YS, Ohkawa S, Springett GM, Wasan HS, Trask PC, et al. Axitinib plus gemcitabine versus placebo plus gemcitabine in patients with advanced pancreatic adenocarcinoma: a double-blind randomised phase 3 study. Lancet Oncol. 2011; 12:256–62.

72. Kindler HL, Niedzwiecki D, Hollis D, Sutherland S, Schrag D, Hurwitz H, Innocenti F, Mulcahy MF, O'Reilly E, Wozniak TF, Picus J, Bhargava P, Mayer RJ, et al. Gemcitabine plus bevacizumab compared with gemcitabine plus placebo in patients with advanced pancreatic cancer: phase III trial of the Cancer and Leukemia Group B (CALGB 80303). J Clin Oncol. 2010; 28:3617–22.

73. Kindler HL, Richards DA, Garbo LE, Garon EB, Stephenson Jr JJ, Rocha-Lima CM, Safran H, Chan D, Kocs DM, Galimi F, McGreivy J, Bray SL, Hei Y, et al. A randomized, placebo-controlled phase 2 study of ganitumab (AMG 479) or conatumumab (AMG 655) in combination with gemcitabine in patients with metastatic pancreatic cancer. Ann Oncol. 2012; 23:2834–42.

74. Kindler HL, Wroblewski K, Wallace JA, Hall MJ, Locker G, Nattam S, Agamah E, Stadler WM, Vokes EE. Gemcitabine plus sorafenib in patients with advanced pancreatic cancer: a phase II trial of the University of Chicago Phase II Consortium. Invest New Drugs. 2012; 30:382–6.

75. Ko AH, Dito E, Schillinger B, Venook AP, Bergsland EK, Tempero MA. Phase II study of fixed dose rate gemcitabine with cisplatin for metastatic adenocarcinoma of the pancreas. J Clin Oncol. 2006; 24:379–85.

76. Kordes S, Pollak MN, Zwinderman AH, Mathôt RA, Weterman MJ, Beeker A, Punt CJ, Richel DJ, Wilmink JW. Metformin in patients with advanced pancreatic cancer: a double-blind, randomised, placebo-controlled phase 2 trial. Lancet Oncol. 2015; 16:839–47.

77. Kulke MH, Tempero MA, Niedzwiecki D, Hollis DR, Kindler HL, Cusnir M, Enzinger PC, Gorsch SM, Goldberg RM, Mayer RJ. Randomized phase II study of gemcitabine administered at a fixed dose rate or in combination with cisplatin, docetaxel, or irinotecan in patients with metastatic pancreatic cancer: CALGB 89904. J Clin Oncol. 2009; 27:5506–12.

78. Kullmann F, Hollerbach S, Dollinger MM, Harder J, Fuchs M, Messmann H, Trojan J, Gäbele E, Hinke A, Hollerbach C, Endlicher E. Cetuximab plus gemcitabine/oxaliplatin (GEMOXCET) in first-line metastatic pancreatic cancer: a multicentre phase II study. Br J Cancer. 2009; 100:1032–6.

79. Kurtz JE, Négrier S, Husseini F, Limacher JM, Borel C, Wagner JP, Prévot G, Bergerat JP, Dufour P. A phase II study of docetaxel-irinotecan combination in advanced pancreatic cancer. Hepato-gastroenterology. 2003; 50:567–70.

80. Lima CM, Lin EH, Kim GP, Giguere JK, Marshall J, Zalupski M, Papageorgio C, Auber ML, Kaleta R, McHenry MB, Trifan OC, Philip PA. A phase 2 trial of ixabepilone plus cetuximab in first-line treatment of metastatic pancreatic cancer. Gastrointest Cancer Res. 2012; 5:155–60.

81. Louvet C, Andre T, Hammel P, Selle F, Landi B, Cattan S, Fonck M, Flesch M, Colin P, Balosso J, Ruszniewski P, de Gramont A. Phase II trial of bimonthly leucovorin, 5-fluorouracil and gemcitabine for advanced pancreatic adenocarcinoma (FOLFUGEM). Ann Oncol. 2001; 12:675–9.

82. Louvet C, André T, Lledo G, Hammel P, Bleiberg H, Bouleuc C, Gamelin E, Flesch M, Cvitkovic E, de Gramont A. Gemcitabine combined with oxaliplatin in advanced pancreatic adenocarcinoma: final results of a GERCOR multicenter phase II study. J Clin Oncol. 2002; 20:1512–8.

83. Louvet C, Labianca R, Hammel P, Lledo G, Zampino MG, Andre T, Zaniboni A, Ducreux M, Aitini E, Taieb J, Faroux R, Lepere C, de Gramont A. Gemcitabine in combination with oxaliplatin compared with gemcitabine alone in locally advanced or metastatic pancreatic cancer: results of a GERCOR and GISCAD phase III trial. J Clin Oncol. 2005; 23:3509–16.

84. Lutz MP, Cutsem EV, Wagener T, Van Laethem JL, Vanhoefer U, Wils JA, Gamelin E, Koehne CH, Arnaud JP, Mitry E, Husseini F, Reichardt P, El-Serafi M, et al. Docetaxel plus gemcitabine or docetaxel plus cisplatin in advanced pancreatic carcinoma: randomized phase II study 40984 of the European Organisation for Research and Treatment of Cancer Gastrointestinal Group. J Clin Oncol. 2005; 23:9250–6.

85. Lutz MP, Königer M, Muche R, Ellenrieder V, Steinkamp M, Adler G, Gress TM. A phase II study of weekly 24-h infusion of high-dose 5-fluorouracil in advanced pancreatic cancer. Z Gastroenterology. 1999; 37:993–7.

86. Mackenzie MJ, Saltman D, Hirte H, Low J, Johnson C, Pond G, Moore MJ. A Phase II study of 3-aminopyridine-2-carboxaldehyde thiosemicarbazone (3-AP) and gemcitabine in advanced pancreatic carcinoma. A trial of the Princess Margaret hospital Phase II consortium. Invest New Drugs. 2007; 25:553–8.

87. Mani S, Kugler JW, Sciortino DF, Garcia JC, Ansari RH, Humerickhouse R, Michelassi F, Posner MC, Shulman KL, Schilsky RL, List M, Vokes EE, Benner S. Phase II trial of uracil/tegafur (UFT) plus leucovorin in patients with advanced pancreatic carcinoma: a University of Chicago phase II consortium study. Ann Oncol. 1998; 9:1035–7.

88. Martin LK, Li X, Kleiber B, Ellison EC, Bloomston M, Zalupski M, Bekaii-Saab TS. VEGF remains an interesting target in advanced pancreas cancer (APCA): results of a multi-institutional phase II study of bevacizumab, gemcitabine, and infusional 5-fluorouracil in patients with APCA. Ann Oncol. 2012; 23:2812–20.

89. Middleton G, Silcocks P, Cox T, Valle J, Wadsley J, Propper D, Coxon F, Ross P, Madhusudan S, Roques T, Cunningham D, Falk S, Wadd N, et al. Gemcitabine and capecitabine with or without telomerase peptide vaccine GV1001 in patients with locally advanced or metastatic pancreatic cancer (TeloVac): an open-label, randomised, phase 3 trial. Lancet Oncol. 2014; 15:829–40.

90. Miller KD, Picus J, Blanke C, John W, Clark J, Shulman LN, Thornton D, Rowinsky E, Loehrer PJ. Phase II study of the multitargeted antifolate LY231514 (ALIMTA™, MTA, pemetrexed disodium) in patients with advanced pancreatic cancer. Ann Oncol. 2000; 11:101–3.

91. Mitry E, Hammel P, Deplanque G, Mornex F, Levy P, Seitz JF, Moussy A, Kinet JP, Hermine O, Rougier P, Raymond E. Safety and activity of masitinib in combination with gemcitabine in patients with advanced pancreatic cancer. Cancer Chemother Pharmacol. 2010; 66:395–403.

92. Moore MJ, Goldstein D, Hamm J, Figer A, Hecht JR, Gallinger S, Au HJ, Murawa P, Walde D, Wolff RA, Campos D, Lim R, Ding K, et al. Erlotinib plus gemcitabine compared with gemcitabine alone in patients with advanced pancreatic cancer: a phase III trial of the National Cancer Institute of Canada Clinical Trials Group. J Clin Oncol. 2007; 25:1960–6.

93. Moore MJ, Hamm J, Dancey J, Eisenberg PD, Dagenais M, Fields A, Hagan K, Greenberg B, Colwell B, Zee B, Tu D, Ottaway J, Humphrey R, et al. Comparison of gemcitabine versus the matrix metalloproteinase inhibitor BAY 12-9566 in patients with advanced or metastatic adenocarcinoma of the pancreas: a phase III trial of the National Cancer Institute of Canada Clinical Trials Group. J Clin Oncol. 2003; 21:3296–302.

94. Moss RA, Moore D, Mulcahy MF, Nahum K, Saraiya B, Eddy S, Kleber M, Poplin EA. A multi-institutional phase 2 study of imatinib mesylate and gemcitabine for first-line treatment of advanced pancreatic cancer. Gastrointest Cancer Res. 2012; 5:77–83.

95. Nakamura K, Yamaguchi T, Ishihara T, Sudo K, Kato H, Saisho H. Phase II trial of oral S-1 combined with gemcitabine in metastatic pancreatic cancer. Br J Cancer. 2006; 94:1575–9.

96. Novarino A, Chiappino I, Bertelli GF, Heouaine A, Ritorto G, Addeo A, Bellone G, Merlano M, Bertetto O. Phase II study of cisplatin, gemcitabine and 5-fluorouracil in advanced pancreatic cancer. Ann Oncol. 2004; 15:474–7.

97. Oettle H, Arning M, Pelzer U, Arnold D, Stroszczynski C, Langrehr J, Reitzig P, Kindler M, Herrenberger J, Musch R, Korsten FW, Huhn D, Riess H. A phase II trial of gemcitabine in combination with 5-fluorouracil (24-hour)

and folinic acid in patients with chemonaive advanced pancreatic cancer. Ann Oncol. 2000; 11:1267–72.

98. Oettle H, Richards D, Ramanathan RK, Van Laethem JL, Peeters M, Fuchs M, Zimmermann A, John W, Von Hoff D, Arning M, Kindler HL. A phase III trial of pemetrexed plus gemcitabine versus gemcitabine in patients with unresectable or metastatic pancreatic cancer. Ann Oncol. 2005; 16:1639–45.
99. Okada S, Sakata Y, Matsuno S, Kurihara M, Sasaki Y, Ohashi Y, Taguchi T. Phase II study of docetaxel in patients with metastatic pancreatic cancer: a Japanese cooperative study. Br J Cancer. 1999; 80:438–43.
100. Okusaka T, Funakoshi A, Furuse J, Boku N, Yamao K, Ohkawa S, Saito H. A late phase II study of S-1 for metastatic pancreatic cancer. Cancer Chemother Pharmacol. 2008; 61:615–21.
101. Okusaka T, Ishii H, Funakoshi A, Ueno H, Furuse J, Sumii T. A phase I/II study of combination chemotherapy with gemcitabine and 5-fluorouracil for advanced pancreatic cancer. Jpn J Clin Oncol. 2006; 36:557–63.
102. Pazdur R, Ajani JJ, Abbruzzese JL, Belt RJ, Dakhil SR, Dubovsky D, Graham S, Pilat S, Winn R, Levin B. Phase II Evaluation of Fluorouracil and Recombinant α-2a-Interferon in Previously Untreated Patients with Pancreatic Adenocarcinoma. Cancer. 1992; 70:2073–6.
103. Pedersen KS, Kim GP, Foster NR, Wang-Gillam A, Erlichman C, McWilliams RR. Phase II trial of gemcitabine and tanespimycin (17AAG) in metastatic pancreatic cancer: a Mayo Clinic Phase II Consortium study. Invest New Drugs. 2015; 33:963–8.
104. Petty RD, Nicolson MC, Skaria S, Sinclair TS, Samuel LM, Koruth M. A phase II study of mitomycin C, cisplatin and protracted infusional 5-fluorouracil in advanced pancreatic carcinoma: efficacy and low toxicity. Ann Oncol. 2003; 14:1100–5.
105. Philip PA, Benedetti J, Corless CL, Wong R, O'Reilly EM, Flynn PJ, Rowland KM, Atkins JN, Mirtsching BC, Rivkin SE, Khorana AA, Goldman B, Fenoglio-Preiser CM, et al. Phase III study comparing gemcitabine plus cetuximab versus gemcitabine in patients with advanced pancreatic adenocarcinoma: Southwest Oncology Group–directed intergroup trial S0205. J Clin Oncol. 2010; 28:3605–10.
106. Philip PA, Goldman B, Ramanathan RK, Lenz HJ, Lowy AM, Whitehead RP, Wakatsuki T, Iqbal S, Gaur R, Benedetti JK, Blanke CD. Dual blockade of epidermal growth factor receptor and insulin-like growth factor receptor–1 signaling in metastatic pancreatic cancer: Phase Ib and randomized phase II trial of gemcitabine, erlotinib, and cixutumumab versus gemcitabine plus erlotinib (SWOG S0727). Cancer. 2014; 120:2980–5.
107. Poplin E, Feng Y, Berlin J, Rothenberg ML, Hochster H, Mitchell E, Alberts S, O'Dwyer P, Haller D, Catalano P, Cella D, Benson III AB). Phase III, randomized study of gemcitabine and oxaliplatin versus gemcitabine (fixed-dose rate infusion) compared with gemcitabine (30-minute infusion) in patients with pancreatic carcinoma E6201: a trial of the Eastern Cooperative Oncology Group. J Clin Oncol. 2009; 27:3778–85.
108. Raderer M, Kornek GV, Hejna MH, Weinlaender G, Vorbeck F, Fiebiger WC, Scheithauer W. Treatment of advanced pancreatic cancer with epirubicin, 5-fluorouracil and l-leucovorin: A phase II study. Ann Oncol. 1997; 8:797–9.
109. Reni M, Passoni P, Panucci MG, Nicoletti R, Galli L, Balzano G, Zerbi A, Di Carlo V, Villa E. Definitive results of a phase II trial of cisplatin, epirubicin, continuous-infusion fluorouracil, and gemcitabine in stage IV pancreatic adenocarcinoma. J Clin Oncol. 2001; 19:2679–86.
110. Renouf DJ, Moore MJ, Hedley D, Gill S, Jonker D, Chen E, Walde D, Goel R, Southwood B, Gauthier I, Walsh W, McIntosh L, Seymour L. A phase I/II study of the Src inhibitor saracatinib (AZD0530) in combination with gemcitabine in advanced pancreatic cancer. Invest New Drugs. 2012; 30:779–86.
111. Richards DA, Boehm KA, Waterhouse DM, Wagener DJ, Krishnamurthi SS, Rosemurgy A, Grove W, Macdonald K, Gulyas S, Clark M, Dasse KD. Gemcitabine plus CI-994 offers no advantage over gemcitabine alone in the treatment of patients with advanced pancreatic cancer: results of a phase II randomized, double-blind, placebo-controlled, multicenter study. Ann Oncol. 2006; 17:1096–102.
112. Rocha Lima CM, Green MR, Rotche R, Miller Jr WH, Jeffrey GM, Cisar LA, Morganti A, Orlando N, Gruia G, Miller LL. Irinotecan plus gemcitabine results in no survival advantage compared with gemcitabine monotherapy in patients with locally advanced or metastatic pancreatic cancer despite increased tumor response rate. J Clin Oncol. 2004; 22:3776–83.
113. Rothman H, Cantrell JE, Lokich J, Difino S, Harvey J, Ahlgren J, Fryer J. Continuous infusion 5-fluorouracil plus weekly cisplatin for pancreatic carcinoma. A mid-atlantic oncology program study. Cancer. 1991; 68:264–8.
114. Rougier P, Adenis A, Ducreux M, De Forni M, Bonneterre J, Dembak M, Clouet P, Lebecq A, Baille P, Lefresne-Soulas F, Blanc C, Armand JP. A phase II study: docetaxel as first-line chemotherapy for advanced pancreatic adenocarcinoma. Eur J Cancer. 2000; 36:1016–25.
115. Ryan DP, Kulke MH, Fuchs CS, Grossbard ML, Grossman SR, Morgan JA, Earle CC, Shivdasani R, Kim H, Mayer RJ, Clark JW. A phase II study of gemcitabine and docetaxel in patients with metastatic pancreatic carcinoma. Cancer. 2002; 94:97–103.
116. Saif MW, Oettle H, Vervenne WL, Thomas JP, Spitzer G, Visseren-Grul C, Enas N, Richards DA. Randomized double-blind phase II trial comparing gemcitabine plus LY293111 versus gemcitabine plus placebo in advanced adenocarcinoma of the pancreas. Cancer J. 2009; 15:339–43.
117. Scheithauer W, Kornek GV, Raderer M, Hejna M, Valencak J, Miholic J, Kovats E, Lang F, Funovics J, Bareck E, Depisch D. Phase II trial of gemcitabine, epirubicin and

granulocyte colony-stimulating factor in patients with advanced pancreatic adenocarcinoma. Br J Cancer. 1999; 80:1797–802.

118. Scheithauer W, Pfeffel F, Kornek G, Marczell A, Wiltschke C, Funovics J. A phase II trial of 5-fluorouracil, leucovorin, and recombinant alpha-2b-interferon in advanced adenocarcinoma of the pancreas. Cancer. 1992; 70:1864–6.

119. Scheithauer W, Schüll B, Ulrich-Pur H, Schmid K, Raderer M, Haider K, Kwasny W, Depisch D, Schneeweiss B, Lang F, Kornek GV. Biweekly high-dose gemcitabine alone or in combination with capecitabine in patients with metastatic pancreatic adenocarcinoma: a randomized phase II trial. Ann Oncol. 2003; 14:97–104.

120. Scher RM, Kosierowski R, Lusch C, Alexander R, Fox S, Redei I, Green F, Raskay B, Amfoh K, Engstrom PF, O'Dwyer PJ. Phase II trial of topotecan in advanced or metastatic adenocarcinoma of the pancreas. Invest New Drugs. 1995; 13:347–54.

121. Schneider BP, Ganjoo KN, Seitz DE, Picus J, Fata F, Stoner C, Calley C, Loehrer PJ. Phase II study of gemcitabine plus docetaxel in advanced pancreatic cancer: a Hoosier Oncology Group study. Oncology. 2003; 65:218–23.

122. Shepard RC, Levy DE, Berlin JD, Stuart K, Harris JE, Aviles V, Thomas JP. Phase II study of gemcitabine in combination with docetaxel in patients with advanced pancreatic carcinoma (E1298). Oncology. 2004; 66:303–9.

123. Spano JP, Chodkiewicz C, Maurel J, Wong R, Wasan H, Barone C, Létourneau R, Bajetta E, Pithavala Y, Bycott P, Trask P, Liau K, Ricart AD, et al. Efficacy of gemcitabine plus axitinib compared with gemcitabine alone in patients with advanced pancreatic cancer: an open-label randomised phase II study. Lancet. 2008; 371:2101–8.

124. Sparano JA, Lipsitz S, Wadler S, Hansen R, Bushunow PW, Kirkwood J, Flynn PJ, Dutcher JP, Benson AB. Phase II trial of prolonged continuous infusion of 5-fluorouracil and interferon-α in patients with advanced pancreatic cancer: Eastern cooperative oncology group protocol 3292. Am J Clin Oncol. 1996; 19:546–51.

125. Sporn JR, Buzaid AC, Slater D, Cohen N, Greenberg BR. Treatment of advanced pancreatic adenocarcinoma with 5-FU, leucovorin, interferon-α-2b, and cisplatin. Am J Clin Oncol. 1997; 20:81–3.

126. Stathopoulos GP, Mavroudis D, Tsavaris N, Kouroussis C, Aravantinos G, Agelaki S, Kakolyris S, Rigatos SK, Karabekios S, Georgoulias V. Treatment of pancreatic cancer with a combination of docetaxel, gemcitabine and granulocyte colony-stimulating factor: a phase II study of the Greek Cooperative Group for Pancreatic Cancer. Ann Oncol. 2001; 12:101–3.

127. Stathopoulos GP, Rigatos SK, Dimopoulos MA, Giannakakis T, Foutzilas G, Kouroussis C, Janninis D, Aravantinos G, Androulakis N, Agelaki S, Stathopoulos JG, Georgoulias V. Treatment of pancreatic cancer with a combination of irinotecan (CPT-11) and gemcitabine: a multicenter phase II study by the Greek Cooperative Group for Pancreatic Cancer. Ann Oncol. 2003; 143:388–94.

128. Stathopoulos GP, Syrigos K, Aravantinos G, Polyzos A, Papakotoulas P, Fountzilas G, Potamianou A, Ziras N, Boukovinas J, Varthalitis J, Androulakis N, Kotsakis A, Samonis G, et al. A multicenter phase III trial comparing irinotecan-gemcitabine (IG) with gemcitabine (G) monotherapy as first-line treatment in patients with locally advanced or metastatic pancreatic cancer. Br J Cancer. 2006; 95:587–92.

129. Stathopoulos GP, Syrigos K, Polyzos A, Fountzilas G, Rigatos SK, Ziras N, Potamiannou A, Tsiakopoulos I, Androulakis N, Aravantinos G, Athanasiadis A, Papakotoulas P, Georgoulias V. Front-line treatment of inoperable or metastatic pancreatic cancer with gemcitabine and capecitabine: an intergroup, multicenter, phase II study. Ann Oncol. 2004; 15:224–9.

130. Stein SM, James ES, Deng Y, Cong X, Kortmansky JS, Li J, Staugaard C, Indukala D, Boustani AM, Patel V, Cha CH, Salem RR, Chang B, et al. Final analysis of a phase II study of modified FOLFIRINOX in locally advanced and metastatic pancreatic cancer. Br J Cancer. 2016; 114:737–43.

131. Taieb J, Lecomte T, Aparicio T, Asnacios A, Mansourbakht T, Artru P, Fallik D, Spano JP, Landi B, Lledo G, Desrame J. FOLFIRI. 3, a new regimen combining 5-fluorouracil, folinic acid and irinotecan, for advanced pancreatic cancer: results of an Association des Gastro-Enterologues Oncologues (Gastroenterologist Oncologist Association) multicenter phase II study. Ann Oncol. 2006; 18:498–503.

132. Tempero M, Plunkett W, Ruiz van Haperen V, Hainsworth J, Hochster H, Lenzi R, Abbruzzese J. Randomized phase II comparison of dose-intense gemcitabine: thirty-minute infusion and fixed dose rate infusion in patients with pancreatic adenocarcinoma. J Clin Oncol. 2003; 21:3402–8.

133. Trouilloud I, Dupont-Gossard AC, Malka D, Artru P, Gauthier M, Lecomte T, Aparicio T, Thirot-Bidault A, Lobry C, Asnacios A, Manet-Lacombe S, Fein F, Dubreuil O, et al. Fixed-dose rate gemcitabine alone or alternating with FOLFIRI. 3 (irinotecan, leucovorin and fluorouracil) in the first-line treatment of patients with metastatic pancreatic adenocarcinoma: An AGEO randomised phase II study (FIRGEM). Eur J Cancer. 2014; 50:3116–24.

134. Tsavaris N, Tentas K, Tzivras M, Kosmas C, Kalachanis N, Katsikas M, Dimitrakopoulos A, Papastratis G, Macheras A, Karatzas G, Sechas M. Combined epirubicin, 5-fluorouracil and folinic acid vs no treatment for patients with advanced pancreatic cancer: a prospective comparative study. J Chemother. 1998; 10:331–7.

135. Ueno H, Okada S, Okusaka T, Ikeda M, Kuriyama H. Phase II study of uracil-tegafur in patients with metastatic pancreatic cancer. Oncology. 2002; 62:223–7.

136. Ueno H, Okusaka T, Ikeda M, Ishiguro Y, Morizane C, Matsubara J, Furuse J, Ishii H, Nagase M, Nakachi K. Phase II study of combination chemotherapy with gemcitabine and cisplatin for patients with metastatic pancreatic cancer. Jpn J Clin Oncol. 2007; 37:515–20.

137. Ueno H, Okusaka T, Ikeda M, Takezako Y, Morizane C. An early phase II study of S-1 in patients with metastatic pancreatic cancer. Oncology. 2005; 68:171–8.
138. Ulrich-Pur H, Kornek GV, Raderer M, Haider K, Kwasny W, Depisch D, Greul R, Schneeweiss B, Krauss G, Funovics J, Scheithauer W. A phase II trial of biweekly high dose gemcitabine for patients with metastatic pancreatic adenocarcinoma. Cancer. 2000; 88:2505–11.
139. Van Cutsem E, Li CP, Nowara E, Aprile G, Moore M, Federowicz I, Van Laethem JL, Hsu C, Tham CK, Stemmer SM, Lipp R, Zeaiter A, Fittipaldo A, et al. Dose escalation to rash for erlotinib plus gemcitabine for metastatic pancreatic cancer: the phase II RACHEL study. Br J Cancer. 2014; 111:2067–75.
140. Van Cutsem E, Van De Velde H, Karasek P, Oettle H, Vervenne WL, Szawlowski A, Schoffski P, Post S, Verslype C, Neumann H, Safran H, Humblet Y, Ruixo JP, et al. Phase III trial of gemcitabine plus tipifarnib compared with gemcitabine plus placebo in advanced pancreatic cancer. J Clin Oncol. 2004; 22:1430–8.
141. Van Cutsem E, Vervenne WL, Bennouna J, Humblet Y, Gill S, Van Laethem JL, Verslype C, Scheithauer W, Shang A, Cosaert J, Moore MJ. Phase III trial of bevacizumab in combination with gemcitabine and erlotinib in patients with metastatic pancreatic cancer. J Clin Oncol. 2009; 27:2231–7.
142. Von Hoff DD, Ramanathan RK, Borad MJ, Laheru DA, Smith LS, Wood TE, Korn RL, Desai N, Trieu V, Iglesias JL, Zhang H, Soon-Shiong P, Shi T, et al. Gemcitabine plus nab-paclitaxel is an active regimen in patients with advanced pancreatic cancer: a phase I/II trial. J Clin Oncol. 2011; 29:4548–54.
143. Wagener DT, Verdonk HE, Dirix LY, Catimel G, Siegenthaler P, Buitenhuis M, Mathieu-Boue A, Verweij J. Phase II trial of CPT-11 in patients with advanced pancreatic cancer, an EORTC early clinical trials group study. Ann Oncol. 1995; 6:129–32.
144. Wagner AD, Buechner-Steudel P, Wein A, Schmalenberg H, Lindig U, Moehler M, Behrens R, Kleber G, Kuss O, Fleig WE. Gemcitabine, oxaliplatin and weekly high-dose 5-FU as 24-h infusion in chemonaive patients with advanced or metastatic pancreatic adenocarcinoma: a multicenter phase II trial of the Arbeitsgemeinschaft Internistische Onkologie (AIO). Ann Oncol. 2006; 18:82–7.
145. Weinerman BH, MacCormick RE. A phase II survival comparison of patients with adenocarcinoma of the pancreas treated with 5-fluorouracil and calcium leucovorin versus a matched tumor registry control population. Am J Clin Oncol. 1994; 17:467–9.
146. Whitehead RP, Jacobson J, Brown TD, Taylor SA, Weiss GR, Macdonald JS. Phase II trial of paclitaxel and granulocyte colony-stimulating factor in patients with pancreatic carcinoma: a Southwest Oncology Group study. J Clin Oncol. 1997; 15:2414–9.
147. Wils J, Bleiberg H, Buyse M, Wagener DT, Splinter T, Veenhof C, Herben M, Duez N. An EORTC Gastrointestinal Group phase II evaluation of epirubicin combined with ifosfamide in advanced adenocarcinoma of the pancreas. Eur J Cancer Clin Oncol. 1989; 25:1119–20.
148. Wu C, Fernandez SA, Criswell T, Chidiac TA, Guttridge D, Villalona-Calero M, Bekaii-Saab TS. Disrupting cytokine signaling in pancreatic cancer: A phase I/II study of Etanercept in combination with Gemcitabine in patients with advanced disease. Pancreas. 2013; 42:813.
149. Xiong HQ, Rosenberg A, LoBuglio A, Schmidt W, Wolff RA, Deutsch J, Needle M, Abbruzzese JL. Cetuximab, a monoclonal antibody targeting the epidermal growth factor receptor, in combination with gemcitabine for advanced pancreatic cancer: a multicenter phase II Trial. J Clin Oncol. 2004; 22:2610–6.
150. Xiros N, Papacostas P, Economopoulos T, Samelis G, Efstathiou E, Kastritis E, Kalofonos H, Onyenadum A, Skarlos D, Bamias A, Gogas H, Bafaloukos D, Samantas E, et al. Carboplatin plus gemcitabine in patients with inoperable or metastatic pancreatic cancer: a phase II multicenter study by the Hellenic Cooperative Oncology Group. Ann Oncol. 2005; 16:773–9.
151. Wils J, Bleiberg H, Dalesio O, Duez N, Blijham G, Planting A, Splinter T, Weber W. An EORTC gastrointestinal group Phase II evaluation of epirubicin combined with 5-fluorouracil in advanced adenocarcinoma of the pancreas. Eur J Cancer Clin Oncol. 1987; 23:1017–8.
